# Perinatal and postnatal risk factors for disruptive mood dysregulation disorder at age 11: 2004 Pelotas Birth Cohort Study

**DOI:** 10.1016/j.jad.2017.03.040

**Published:** 2017-06

**Authors:** Tiago N. Munhoz, Iná S. Santos, Aluísio J.D. Barros, Luciana Anselmi, Fernando C. Barros, Alicia Matijasevich

**Affiliations:** a Postgraduate Program in Epidemiology, Federal University of Pelotas, Pelotas, Brazil; b Faculty of Psychology, Federal University of Pelotas, Pelotas, Brazil; c Health and Behavior Postgraduate Program, Catholic University of Pelotas, Pelotas, RS, Brazil; d Department of Preventive Medicine, Faculty of Medicine, University of São Paulo, São Paulo, Brazil

**Keywords:** Adolescent psychiatry, Mood disorders, Neurodevelopmental disorders, Mental disorders diagnosed in childhood, Epidemiology, Mental disorders

## Abstract

**Background:**

To date, there have been few studies of DMDD examining the risk factors during gestation and during the first years of life. We assessed the perinatal and postnatal risk factors associated with the occurrence of disruptive mood dysregulation disorder (DMDD) by 11 years of age.

**Methods:**

Prospective longitudinal study. Mothers completed a standardized questionnaire shortly after childbirth. We used the Development and Well-Being Assessment, administered to the mothers or legal guardians, to identify DMDD among the 11-year-old subjects. We also employed logistic regression to perform bivariate and multivariate analyses, using a theoretical model of conceptual analysis.

**Results:**

We evaluated data related to 3563 subjects at 11 years of age. The prevalence of DMDD was 2.5% (95% CI=2.0–3.0). After adjusting for potential confounders, we found that the early risk factors for the development of DMDD by 11 years of age were maternal mood symptoms during pregnancy, maternal depression during the first years after childbirth, and low maternal level of education.

**Limitations:**

We were unable to evaluate the genetic characteristics of the family at the birth of each subject, and there were no data available regarding the prenatal or postnatal mental health of the fathers.

**Conclusions:**

The prevalence of DMDD in early adolescence is low and its risk factors are related to potentially modifiable maternal characteristics. Scientific evidence indicates that DMDD is a major predictor of other psychiatric disorders, especially depression and anxiety. Effective prenatal and postnatal mental health care could prevent mental disorders in offspring.

## Highlights

• DMDD is characterized by irritability and persistent outbursts of anger.
• DMDD is a new diagnosis included in DSM-5.
• The prevalence of DMDD in early adolescence is low.
• DMDD risk factors are related to potentially modifiable maternal characteristics.

## 1 Introduction

Disruptive mood dysregulation disorder (DMDD) is characterized by irritability and persistent outbursts of anger that are inappropriate for the developmental stage. Those symptoms result in a pattern of chronic irritable behavior. Such symptoms manifest predominantly as verbal attacks and physical aggression directed at family members, colleagues, and teachers. The DSM-5 considers the diagnosis of DMDD only in individuals under 18 years of age.

In the field of psychiatric epidemiology, there is a consensus that mental disorders often begin in childhood or adolescence and persist into adult life, potentially even affecting the well-being of subsequent generations. In a systematic review, Kieling et al. (Kieling et al., 2011) noted that the prevalence of mental disorders in childhood and adolescence ranges from 1.8% to 39.4% in low- and middle-income countries, attributing that variation to differences among the studies evaluated, in terms of the instruments and methodology employed. They also observed that the risk factors and protective factors of mental disorders in childhood and adolescence include genetic and environmental characteristics.

To date, there are few studies examining early risk factors for DMDD (Vidal-Ribas et al., 2016) and the current evidence suggests that the risk of developing DMDD is higher among the children of parents with psychopathology than among the other children (Sparks et al., 2014, Tufan et al., 2016) and no other familial or social characteristics associated with DMDD have been previously investigated. In a systematic review with meta-analysis, Vidal-Ribas et al. (Vidal-Ribas et al., 2016) showed that the irritability dimension of DMDD was distinct from that of oppositional defiant disorder (ODD). The authors found that the risk of depression and anxiety in later life is higher for individuals who, during childhood, show symptoms of DMDD-like irritability or are diagnosed with DMDD, as well as that the persistence of symptoms of irritability is associated with altered activation of the amygdala, striatum, and frontal regions of the brain. It has also been demonstrated that a parental history of bipolar disorder increases the risk of developing DMDD in childhood and adolescence (Sparks et al., 2014). There is evidence of a difference between men and women in terms of the pattern of genetic effects on irritability, the genetic influence being greater among women only during childhood (Roberson-Nay et al., 2015).

Long-term consequences among those diagnosed with DMDD during childhood included higher risk of developing depressive disorders by the age of 19, (Brotman et al., 2006) poorer academic performance, increased use of health care services, and lower socioeconomic status in adulthood (Copeland et al., 2013). There have been few population-based studies of DMDD in low- and middle-income countries (Tufan et al., 2016). Therefore, the objective of the present study was to evaluate the perinatal and postnatal risk factors associated with the occurrence of DMDD by 11 years of age in a middle-income country.

## 2 Methods

### 2.1 Participants

In 2004, we invited all women whose children were born in the city of Pelotas, Brazil, to participate in a study, designated the 2004 Pelotas Birth Cohort Study. Pelotas is in the south of Brazil and has a population of approximately 328,000, of whom 93.3% live in the urban part of the city. In Pelotas, more than 99% of all births take place in hospitals. We identified all births occurring in 2004 by making daily visits to all of the maternity hospitals in the city. Mothers were interviewed shortly after the birth of the child. We collected data related to demographic, socioeconomic, behavioral, and biological characteristics, as well as reproductive history and the previous use of health care services. The interviews were conducted by trained interviewers. During perinatal follow-up, the response rate was above 99%. A detailed description of the study design can be found elsewhere (Santos et al., 2011, Santos et al., 2014). A total of 4231 live births were included in the cohort. Follow-up home visits were conducted when the subjects had, on average, reached the ages of 3.0 months (SD=0.1), 11.9 months (SD=0.2), 23.9 months (SD=0.4), and 49.5 months (SD=1.7). When the subjects were, on average, 6.8 years of age (SD=0.3) and 11.0 years of age (SD=0.4), additional follow-up visits were conducted, at a clinic built especially for the study. During the follow-up visits, attendance rates ranged from 87% to 96%.

### 2.2 Outcome

To diagnose DMDD, we used the Development and Well-Being Assessment (DAWBA) questionnaire (Goodman et al., 2000). The DAWBA consists of open and closed questions identifying the occurrence of symptoms based on the DSM and ICD diagnostic criteria for mental disorders. It was developed for use in children and adolescents between 5 and 17 years of age. The open questions allow qualitative description of the symptoms, frequency, and other characteristics of the disorders assessed. In addition, a clinical evaluator (rater) can evaluate each of the questionnaires individually, integrating the responses to the questions and determining the diagnosis. A second independent rater evaluated 10% of the subjects in the cohort. The DAWBA was validated in Brazil and the agreement between the two clinical raters was 0.93 for any disorder (SE 0.03), 0.91 for internalizing disorders (SE 0.05), and 1.0 for externalizing disorders (Fleitlich-Bilyk and Goodman, 2004).

The DAWBA was applied to mothers or caregivers by psychologists who had been trained in its standardized administration. The training included lectures, role playing, and supervised clinical interviews with pediatric and mental health outpatients at the Federal University of Pelotas, totaling more than 40 h. The DAWBAs were applied via computer, allowing direct input of the data into an online system. Details of the questionnaire can be found online, as well as in other studies (Petresco et al., 2014). For the purposes of this study, we used the section that evaluates the symptoms of DMDD according to the DSM-5 diagnostic criteria.

### 2.3 Covariates

The exposures included in the present study were recorded during the interviews conducted in the maternity hospitals. We evaluated the following demographic and socioeconomic characteristics of the mother at the time of childbirth: age (≤19, 20–34, or ≥34 years of age); years of schooling; family income in the last month (in quintiles); marital status (married/living with a partner or single/divorced/widowed); skin color (White or other); parity (0, 1, or ≥2 children); and body mass index (BMI) before pregnancy (<18.5, 18.5<25.0, 25.0<30.0, or ≥30.0 kg/m^2^). Maternal schooling was collected as a continuous variable and categorized according to the Brazilian Education System. The System is divided into three levels: fundamental (grades 1–8), intermediate (9–11) and higher education (≥12 years of formal education). Because of the small numbers of women without any formal education (0 years) and those with higher education, we opted to combine them with the nearest category available. In addition, we decided to split the 1–8 category because it is very common in the city for women to start the fundamental level and only complete 4 years. Finally, maternal schooling was categorized as 0–4, 5–8 and ≥9 complete school years of formal education. Skin color was chosen as a proxy for ancestral background because miscegenation in Brazil is highly prevalent (Guimarães, 1995) and it is not feasible to classify individuals into different ethnic groups in large-scale studies.

Hypertension during pregnancy was investigated as a dichotomous variable (yes or no). Smoking and alcohol use during pregnancy were self-reported and were evaluated retrospectively at birth. Women were categorized as having smoked during pregnancy if they reported smoking at least one cigarette per day during any trimester, whereas they were categorized as having used alcohol during pregnancy if they reported any alcohol use during any trimester. Mood symptoms during pregnancy were identified on the basis of an affirmative response to the question: “During your pregnancy, did you ever feel depressed or nervous*” We evaluated the following characteristics of the child, at birth: gender; LBW, defined as birth weight <2500 g (yes or no); 5-min Apgar score (<7 or ≥7); and intrauterine growth restriction (IUGR; yes or no) according to Williams et al. (Williams et al., 1982). Newborns whose weight for gestational age was below the 10th percentile were classified as having IUGR.

Maternal depression was assessed at 3 months after delivery, as well as when the child had reached 1, 2, 4, and 6 years of age. To assess maternal depression, we used the self-report Edinburgh Postnatal Depression Scale (EPDS), which has been translated to Portuguese and validated for use in Brazil (Cox et al., 1987, Santos et al., 2007). The EPDS questionnaire comprises 10 questions that assess symptoms of depression in the last seven days. For each question, there are four possible answers, scored from 0 to 3 points. The EPDS was administered to all of the mothers, except at the 3-month follow-up visit, when it was completed by a subsample of 965 mothers whose children were born between October 1 and December 31 (Matijasevich et al., 2015).

### 2.4 Data analysis

To describe the characteristics of the sample and the prevalence of DMDD for each of the exposures evaluated, we performed univariate analyses, with absolute and relative frequencies. We also used logistic regression to perform bivariate and multivariate analyses, the results of which are expressed as odds ratio and 95% CI. We only included in the multivariate model those variables with p<0.20 in the bivariate analysis. Multiple pregnancies (N=42) were excluded.

To estimate the trajectories of maternal depression we used semiparametric group-based modeling, a form of finite mixture modeling proposed by Nagin & Odgers (36), in order to identify the different trajectories of depressive symptoms reported by the mothers on the EPDS in the follow-up visits conducted between 3 months and 6 years after the birth of their children. Because approximately 30% of the mothers scored less than 4 points total on the EPDS at each follow-up visit a censored normal model was adjusted to the data. The choice of the number and format of the trajectories was based on their interpretability and not only on their fit to the model (maximum Bayesian information criterion) (Matijasevich et al., 2015).

A hierarchical model to adjust for potential confounding factors was used in order to determine the risk factors for DMDD (Victora et al., 1997). That model consisted of six levels: demographic and socioeconomic variables related to the mother; variables related to pregnancy; maternal behavioral traits; mood symptoms during pregnancy; characteristics of the child at birth; and the trajectories of maternal depression between 3 months and 6 years after the birth of the child. The model considers the effect of each variable in relation to the outcome, controlling for confounding among variables of the same or higher level. The adjusted analysis included the variables that presented p<0.20 in all levels of the model. The data were analyzed with STATA statistical software, version 12 (StataCorp, College Station, Tex).

### 2.5 Ethical aspects

The study was approved by the Research Ethics Committee of the Federal University of Pelotas School of Medicine and was conducted in accordance with the ethical standards currently in effect. At each stage of the study, the mothers or legal guardians of all subjects gave written informed consent. All had the right to opt out at any time. At 11 years of age, the subjects also gave written informed consent. Those diagnosed with serious mental health problems were referred to the appropriate local health care facilities.

## 3 Results

Of the 4231 subjects in the original cohort, 3490 (82.5%) were included in the final analysis. In comparison with the subjects who dropped out or were lost to follow-up, those who remained in the study until 11 years of age had mothers who were older, had a higher level of education, had a higher income, were living with a companion at the time of birth, had a higher pre-pregnancy BMI, had a lower prevalence of smoking during pregnancy, and were less likely to present with mood symptoms. The final sample also included a smaller proportion of subjects who had been LBW neonates, as well as of subjects who had a 5-min Apgar score <7. The two groups (those included and those excluded) were similar in relation to IUGR, as well as to the following maternal characteristics: skin color, hypertension during pregnancy, parity, and trajectories of depression (data not shown).

Table 1 presents the characteristics of the sample. Approximately one of every five children were born to mothers who were <20 years of age. Of the mothers evaluated, 14.8% had 0–4 years of schooling, 15.5% were single/divorced/widowed, and 62.3% characterized themselves as White. Hypertension during pregnancy was reported by 8.3% of the mothers. Of the mothers evaluated, 39.7% were primiparous and 34.5% were classified as overweight or obese on the basis of the pre-pregnancy BMI. Smoking and alcohol use during pregnancy were reported by 26.9% and 3.2%, respectively. Mood symptoms during pregnancy were reported by 8.3%. At birth, 51.7% of the subjects were male, 8% were LBW neonates, 3.1% presented a 5-min Apgar ≤7, and 11.9% had IUGR. The prevalence of DMDD was 2.5% (95% CI=2.0–3.0).

Table 2 presents the results of the logistic regression. In the crude analysis, the risk of developing DMDD by 11 years of age was found to be inversely associated with maternal level of education and family income. That risk was also higher among the children of smoking mothers, of mothers with depressive symptoms during pregnancy, and of mothers with chronic depression during the first years after childbirth. The risk of DMDD was higher among those children being born with low birth weight. In the multivariate analysis, the children of mothers with only 0–4 years of schooling had a higher chance of developing DMDD than did the children of mothers with ≥9 years of schooling (OR=3.94; 95% CI=2.08–7.44). The risk of developing DMDD by 11 years of age was 2.3 times higher among the children of mothers with mood symptoms during pregnancy (OR=2.34; 95% CI=1.35–4.05) and 4.6 times higher among those of mothers who were chronically depressed during the first years after childbirth (OR=4.59; 95% CI=1.81–11.64). In the multivariate analysis, DMDD was not associated with family income, parity, smoking during pregnancy, alcohol use during pregnancy, child´s sex and birth weight.

## 4 Discussion

In the Pelotas Birth Cohort, the prevalence of DMDD at 11 years of age was 2.5%. After adjusting for potential confounding factors, we found that the early risk factors for the development of DMDD by 11 years of age were maternal mood symptoms during pregnancy, maternal depression during the first years after childbirth, and low maternal level of education.

Copeland et al. (Copeland et al., 2013) evaluated 3258 individuals (ages 2–17) who collectively participated in three different studies conducted in the United States (US) (the Duke Preschool Anxiety Study, the Great Smoky Mountains Study, and the Caring for Children in the Community study). The authors found that the prevalence of DMDD decreased with age, reaching 3.3% at ages 2–5% and 0.8% at ages 9–17. In a sample of 597 individuals (ages 6–18) treated at community mental health centers in the US, the overall prevalence of DMDD was 31% (Freeman et al., 2016). Tufan et al. (Tufan et al., 2016) analyzed 403 individuals, with a mean age of 9 years (SD=2.5), that were treated at a psychiatric clinic in Turkey, finding that 8.9% met the criteria for a diagnosis of DMDD. The criteria for operationalizing the outcome varied across those studies, being defined from the use of different questionnaires and instruments. That was due to the fact that the diagnostic criteria for DMDD were defined and disseminated after those studies had been conducted. For example, Freeman et al. (Freeman et al., 2016) used the ODD section of The Kiddie Schedule for Affective Disorders and Schizophrenia for School-Age Children (based on DSM-IV criteria) in order to assess the duration and impact of the symptoms. None of the three studies from which the Copeland et al. (Copeland et al., 2013) sample were drawn were designed to evaluate DMDD, all three having employed the Child Behavior Checklist (CBCL). Tufan et al. (Tufan et al., 2016) conducted unstructured clinical interviews according to the DSM-IV-TR criteria. Therefore, these studies defined the outcome differently, creating a potential bias in the identification of DMDD, which hinders the comparison of the results. In addition to the differences in terms of the characteristics of the studies—e.g., study site, subject ages, and population (clinical or community)—the differences in terms of the definition of the outcome could explain the differences in prevalence across the studies.

The risk factors associated with patterns of irritability and DMDD have recently been studied. Sparks et al. (Sparks et al., 2014) evaluated 375 individuals (ages 6–17) from the Pittsburgh Bipolar Offspring Study and found that the risk of developing DMDD was eight times higher among the children of parents diagnosed with bipolar disorder than among the other children. In 2620 children from the Swedish Twin Study of Child and Adolescent Development, Roberson-Nay et al. (Roberson-Nay et al., 2015) evaluated the temporal pattern of the effects that genetic and environmental factors have on irritability, as well as their effects in adulthood. The results indicated that the influence of genetic factors was greater than was that of environmental factors, with a difference between the sexes.

Some studies have identified long-term deficits among adolescents diagnosed with DMDD. Deveney et al. (Deveney et al., 2015) evaluated severe mood dysregulation (SMD) in 200 individuals (ages 7–17), over a four-year period, and identified a pattern of stability in the occurrence of symptoms of irritability. Brotman et al. (Brotman et al., 2006) evaluated SMD in 1420 individuals (ages 9–19) from the Great Smoky Mountains Study and observed that those who had SMD by 10 years of age presented a higher risk of developing depressive disorders by the age of 19. Copeland et al. (Copeland et al., 2013) investigated DMDD in 3258 children and adolescents (ages 2–17). The authors found that individuals with DMDD presented greater social deficits, poorer academic performance, increased use of health care services, and lower socioeconomic status. In another longitudinal study, Copeland et al. (Copeland et al., 2014) evaluated 1420 individuals at 10, 19, 21, and 24–26 years of age. Compared with the individuals with no history of mental disorders, those with DMDD were found to be at an increased risk for health problems, financial difficulties, problems with the police, and poor academic performance by 21 years of age. Dougherty et al. (Dougherty et al., 2016) evaluated 473 children (ages 6–9) from a longitudinal study and observed that those diagnosed with DMDD by 6 years of age were more likely to require remedial education, have poorer relationships with their peers, victimize others, perform acts of aggression against teachers, and require treatment (pharmacological or nonpharmacological) by the age of 9.

In the present study, maternal depression during pregnancy and during the first years after childbirth was found to increase the odds of developing DMDD by age 11. Studies evaluating the effects of maternal depression on the mental health of the offspring have demonstrated that maternal depression increases the risk of the offspring developing mental health problems in childhood or adolescence (22, 35, 44–46). Brennan et al. (44) evaluated 4953 mothers and their children, with the aim of determining how the severity, duration, and chronicity of maternal depressive symptoms affect the behavior of the child at 5 years of age. The authors found that greater severity and chronicity of maternal depressive symptoms were associated with an increased risk of behavioral problems in such children. In a longitudinal study involving 151 mothers and children, Maughan et al. (46) found that maternal depression and poor parenting (negative maternal affective states and insensitive parenting behaviors) were predictors of increased emotional dysregulation in children at 4 years of age. Among 4898 families from the Fragile Families and Child Wellbeing Study, conducted in the US, Wiggins et al. (47) evaluated the association between depressive episodes in parents and the risk of CBCL-determined symptoms of irritability in their children at 3, 5, and 9 years of age. The authors found that maternal depression increased the risk and severity of symptoms of irritability in childhood. They also found that paternal depression and alcohol/drug use were risk factors for increased severity of symptoms of irritability. In a longitudinal study conducted in Brazil, Matijasevich et al. (35) evaluated 3585 children from the same population analyzed in the present study. Those authors found that, even after being adjusted for the characteristics of the mother and child, the likelihood of any mental disorder was greater among the children of mothers who had had chronic depression during the first years after childbirth. Krieger et al. (48) evaluated 2512 children between 6 and 12 years of age in Brazil and found that maternal depression increased the risk of the child presenting the irritability dimension of ODD.

This study had some advantages. First, we evaluated a population-based sample, which allowed us to estimate the true frequency of the disease in the population. The study design also allowed us to identify a set of exposures at birth and to diagnose DMDD at 11 years of age. In addition, we conducted face-to-face interviews and employed a diagnostic instrument that has been validated for the evaluation of mental disorders in Brazil. However, our study also has certain limitations. We were unable to evaluate the genetic characteristics of the family at the birth of each subject, and there were no data available regarding the prenatal or postnatal mental health of the fathers. In addition, the study lacks of more detailed information about maternal mental health status, apart from depression symptomatology after delivery. Because of the low frequency of DMDD in the population studied here, the fact that some perinatal and postnatal features were not associated with DMDD might be attributable to the lack of statistical power to identify such associations. That should be taken into consideration in the interpretation of the results.

## 5 Conclusions

The prevalence of DMDD in early adolescence is low. The early risk factors for DMDD include maternal characteristics, such as a low level of education and depression during pregnancy or after childbirth. In the multivariate analysis, DMDD was not associated with any characteristic of the child or of the pregnancy.

The literature indicates that DMDD is a major predictor of other psychiatric disorders, especially depression and anxiety. Therefore, early, appropriate evaluation of symptoms of irritability and early diagnosis of DMDD could reduce the incidence of mental health problems later in life. The treatment of maternal depression should be a major focus of public health policies, because it is important to reduce its adverse consequences, including its effects on the mental health of children and adolescents.

## Figures and Tables

**Table 1:** Sample characteristics, the prevalence of disruptive mood dysregulation disorder at 11 years of follow-up, and statistical associations between the two, in the 2004 Pelotas Birth Cohort Study.

**Variable**	**Distribution**		**DMDD**^a^**prevalence**	
	**N**	**%**	**%**	**p**
Among mothers (at childbirth)				
Age (years)^b^				0.454
≤19	662	19.0	2.6	
20–34	2340	67.1	2.6	
≥34	486	13.9	1.7	
Years of schooling^b^				<0.001
≥9	1513	43.8	1.3	
5–8	1431	41.4	2.5	
0–4	513	14.8	5.9	
Family income (by quintile)^b^				0.014
1 (lowest)	695	19,5	3,5	
2	714	20,0	3,2	
3	709	19,9	2,4	
4	754	21,2	2,1	
5 (highest)	690	19,4	0,9	
Living with a partner^b^				0.846
No	542	15.5	2.6	
Yes	2948	84.5	2.4	
Skin color				0.491
White	2149	62.3	2.6	
Other	1300	37.7	2.2	
Hypertension during pregnancy^b^				0.657
No	3201	91.7	2.5	
Yes	289	8.3	2.1	
Parity (previous pregnancies, n)^b^				0.084
0	1386	39.7	1.8	
1	934	26.8	2.6	
≥2	1169	33.5	3.2	
Pregestational body mass index^b^				0.198
<18.5 kg/m^2^	147	4.5	3.4	
18.5–<25 kg/m^2^	1984	61.0	2.4	
25–<30 kg/m^2^	768	23.6	1.7	
≥30 kg/m^2^	356	10.9	3.7	
Smoking during pregnancy^b^				0.003
No	2.553	73.1	2.0	
Yes	937	26.9	3.7	
Alcohol use during pregnancy^b^				0.172
No	3377	96.8	2.4	
Yes	113	3.2	4.4	
Mood symptoms during pregnancy^b^				<0.001
No	3196	91.7	2.1	
Yes	290	8.3	6.2	
Depression trajectory^b^				<0.001
Low	1111	35.4	0.9	
Moderate-low	1293	41.2	2.5	
Increasing	277	8.8	4.0	
Decreasing	300	9.6	3.3	
High-chronic	160	5.1	7.5	

Among children				
Sex				0.099
Male	1804	51.7	2.9	
Female	1686	48.3	2.0	
Birth weight^b^				0.038
<2500 g	278	8.0	4.3	
≥2500 g	3211	92.0	2.3	
5-min Apgar score^b^				0.671
<7	108	3.1	1.9	
≥7	3364	96.9	2.5	
Intrauterine growth restriction^b^				0.394
No	2805	88.1	2.2	
Yes	378	11.9	2.9	

a Abbreviations: DMDD, disruptive mood dysregulation disorder.

b Data not available for all subject-variable pairs.

**Table 2:** Bivariate and multivariate logistic regression of the association between the sample characteristics and Disruptive mood Dysregulation Mood Disorder in the 2004 Pelotas Birth Cohort Study at 11 years of follow-up.

**Variable**	**Bivariate analysis**		**Multivariate analysis**	
	**OR (95% CI)**	**p**	**OR (95% CI)**	**p**
LEVEL 1				
Maternal years of schooling		<0.001		<0.001
≥9	1		1	
5–8	2.03 (1.16–3.55)		1.71 (0.95; 3.10)	
0–4	4.88 (2.72–8.76)		3.94 (2.08; 7.44)	
Family income (by quintile)		0.006		0.614
1 (lowest)	4.12 (1.67–10.15)		2.21 (0.85; 5.79)	
2	3.76 (1.52–9.29)		2.00 (0.76; 5.26)	
3	2.78 (1.09–7.10)		1.84 (0.70; 4.86)	
4	2.45 (0.95–6.30)		1.88 (0.72; 4.92)	
5 (highest)	1		1	

LEVEL 2				
Parity (previous pregnancies, n)		0.081		0.586
0	1		1	
1	1.44 (0.81–2.53)		1.37 (0.75; 2.50)	
≥2	1.78 (1.06–2.97)		1.21 (0.67; 2.17)	

LEVEL 3				
Smoking during pregnancy		0.004		0.145
No	1		1	
Yes	1.92 (1.24–2.97)		1.41 (0.89; 2.23)	
Alcohol use during pregnancy		0.179		0.374
No	1		1	
Yes	1.88 (0.75–4.74)		1.54 (0.60–3.96)	

LEVEL 4				
Maternal mood symptoms during pregnancy		<0.001		0.002
No	1		1	
Yes	3.04 (1.78–5.19)		2.34 (1.35–4.05)	

LEVEL 5				
Sex of the child		0.093		0.053
Male	1.44 (0.93–2.23)		1.55 (0.99; 2.43)	
Female	1		1	
Birth weight of the child		0.041		0.138
<2500 g	1.91 (1.03–3.56)		1.62 (0.86; 3.07)	
≥2500 g	1		1	

LEVEL 6				
Trajectories of maternal depression (3 months–6 years)		<0.001		0.027
Low	1		1	
Moderate-low	2.79 (1.37–5.71)		2.29 (1.11; 4.73)	
Increasing	4.55 (1.91–10.83)		2.99 (1.22; 7.31)	
Decreasing	3.80 (1.57–9.21)		2.35 (0.92; 5.99)	
High-chronic	8.93 (3.79–21.02)		4.59 (1.81; 11.64)	
